# PET/MRI attenuation estimation in the lung: A review of past, present, and potential techniques

**DOI:** 10.1002/mp.13943

**Published:** 2020-01-01

**Authors:** Joseph Lillington, Ludovica Brusaferri, Kerstin Kläser, Karin Shmueli, Radhouene Neji, Brian F. Hutton, Francesco Fraioli, Simon Arridge, Manuel Jorge Cardoso, Sebastien Ourselin, Kris Thielemans, David Atkinson

**Affiliations:** ^1^ Centre for Medical Imaging University College London London W1W 7TS UK; ^2^ Institute of Nuclear Medicine University College London London NW1 2BU UK; ^3^ Centre for Medical Image Computing University College London London WC1E 7JE UK; ^4^ Magnetic Resonance Imaging Group Department of Medical Physics & Biomedical Engineering University College London London WC1E 6BT UK; ^5^ MR Research Collaborations Siemens Healthcare Limited Frimley GU16 8QD UK; ^6^ School of Biomedical Engineering & Imaging Sciences King's College London London SE1 7EH UK

**Keywords:** attenuation estimation, lung, medical imaging, magnetic resonance imaging, positron emission tomography

## Abstract

Positron emission tomography/magnetic resonance imaging (PET/MRI) potentially offers several advantages over positron emission tomography/computed tomography (PET/CT), for example, no CT radiation dose and soft tissue images from MR acquired at the same time as the PET. However, obtaining accurate linear attenuation correction (LAC) factors for the lung remains difficult in PET/MRI. LACs depend on electron density and in the lung, these vary significantly both within an individual and from person to person. Current commercial practice is to use a single‐valued population‐based lung LAC, and better estimation is needed to improve quantification. Given the under‐appreciation of lung attenuation estimation as an issue, the inaccuracy of PET quantification due to the use of single‐valued lung LACs, the unique challenges of lung estimation, and the emerging status of PET/MRI scanners in lung disease, a review is timely. This paper highlights past and present methods, categorizing them into segmentation, atlas/mapping, and emission‐based schemes. Potential strategies for future developments are also presented.

## 1 Introduction

The combined imaging modality positron emission tomography/magnetic resonance imaging (PET/MRI) offers several advantages over PET/computed tomography (CT). These include a superior soft tissue contrast, reduced radiation exposure, increased functional information (including perfusion and spectroscopy), MRI‐based motion correction, as well as providing approximately simultaneous rather than sequential acquisitions.^1^, ^2^ Using MRI can also circumvent artifacts from the iodinated contrast agents potentially used in CT and the attenuation map mismatch between the often single‐respiratory phase CT, and multi‐respiratory phase PET.^3^, ^4^

To fully exploit the benefits of these hybrid systems, it is essential to be able to obtain quantitative information from the PET images. This requires access to a map of the electron density in order to correct for attenuation of the PET photons. Attenuation can be modelled via the Lambert Beer law, with associated linear attenuation coefficients (LACs).^5^ In stand‐alone PET systems, measurements of LACs can be performed directly from PET transmission scans and should yield ground‐truth LACs. In contrast, PET/CT scanners use CT for faster and less noisy attenuation correction. Here, the LACs determined with 60–80 keV photons from CT are converted, using bilinear scaling, to the corresponding LAC values for the PET 511 keV photons.^6^ Tabulated compositions can be used as an alternative to bilinear scaling, but Martinez and Calzado^7^ found no relevant difference for bones and lung. Alternatives include trilinear scaling,^8^ the quadratic polynomial calibration curve,^9^ and (virtual‐) dual energy CT methods.^10^, ^11^ Although a toroidal transmission source has been demonstrated for evaluation of attenuation coefficient measures,^12^ the limited space within the scanner bore and the need to measure over the whole‐lung volume prevent use of transmission sources during clinical imaging. This view is supported by the work of Bowen et al.^13^ who stated that their developed transmission scan method is not for routine subject imaging, and by the study of Mollet et al.^14^ who noted that the reduced diameter of the PET/MR system would affect the PET count rate performance of the simultaneous emission/transmission scan. As a consequence, MRI is used (at least in part) for determining attenuation coefficients. Such attenuation factors should perhaps be compared to those determined using PET transmission scans as stated by Schramm et al.^15^ However, this is becoming increasingly difficult due to the limited availability of PET transmission scans. Therefore, comparison to CT‐derived LACs is most commonly performed in the literature.

In recent years, a number of review papers have been published describing attenuation estimation.^3^, ^4^, ^16^, ^17^, ^18^, ^19^, ^20^ Keereman et al.^3^ published an overview of the various techniques, categorizing them into MRI derived (template and voxel‐based), transmission, and emission‐based methods. The descriptions were focused on both the brain and whole body, as were those in the review presented by Martinez‐Möller and Nekolla.^4^ Mehranian et al.^16^ also reviewed attenuation estimation methods, summarizing them into segmentation, atlas registration and machine learning, and emission/transmission types. They stated the advantages and disadvantages of each class of technique, again for the brain and whole body. The authors also noted various challenges and potential solutions associated with attenuation correction, for example, MRI truncation compensation, MRI coil and other device attenuation, and optimized MRI data acquisition. To our knowledge, this has been the only review paper to explicitly separate lung attenuation from whole‐body techniques. It categorized methods into those that predict mean lung LACs from mean MRI intensity and/or lung volume, atlas‐based registration and learning schemes, and emission‐based estimation. However, the summary given was not exhaustive, only representing less than one page in total. Remaining reviews did not distinguish the lungs from whole‐body methods. Bezrukov et al.^17^ focused on attenuation estimation using MR data. Hofmann et al.^18^ reviewed methods for the brain and torso (but not the lung specifically). Finally, Wagenknecht et al.^19^ described template, atlas, direct segmentation from T1‐weighted MRI, and sequence‐based approaches to attenuation estimation, using an intermix of both brain and whole‐body results. They stated the advantages and disadvantages of different techniques, along with the challenges of MRI‐based attenuation estimation. From the combined reviews, it is evident that lung attenuation estimation has been significantly under‐represented, in terms of summarizing past, and present techniques, as well as highlighting potential methods and direction for subsequent improvement.

Inaccurate LACs in PET reconstruction can result in PET quantification errors.^21^ Current PET/MRI scanners use constant, population‐based LACs assigned following segmentation of the whole‐lung from MR images. The MRI signal is not directly related to photon attenuation, and therefore predefined values for LACs are used.^19^ This is an approximation because lung tissue exhibits variation both within an individual, between respiratory states, and from person to person. After incorporating the resultant magnetic resonance attenuation correction (MRAC) maps into reconstruction, PET quantification in the form of standard uptake values (SUVs) is well‐known to be affected. For example, Seith et al.^21^ determined that using constant lung LAC values in non‐Time‐of‐Flight (ToF) PET/MRI underestimated mean SUV in lung tissue regions of interest (ROIs) by 9% in the posterior lung region and over‐estimated by 6% in the anterior and middle lung, relative to PET/CT. For pulmonary studies, Holman et al.^22^ quantified the effect on both Standard Uptake Values (SUVs) and various kinetic parameters of using LACs corresponding to a single breathing state to reconstruct ungated PET data, without TOF, finding errors of up to 30%. It is therefore likely that this error would be even larger when using a fixed population value when studying the diseased lung. The authors determined that respiratory induced mismatches (albeit for PET/CT) affect PET lung quantification with the error being influenced by both the type of tracer and the time since tracer administration. It is thus important that improved lung attenuation estimation is achieved. There is potential for PET/MRI to have an increased role in oncology with lung tumors currently forming a significant focus of PET/CT studies.^23^ In addition, there is increased interest in using PET to study pulmonary diseases.^24^ Improved lung attenuation estimation is also highly relevant in both nodular and diffuse lung disease.

Given the under‐appreciation of lung attenuation estimation as an issue, the inaccuracy of PET quantification due to the use of single‐valued lung LACs, the unique challenges of lung estimation (later stated in Section 3), and the emerging status of PET/MRI scanners in both nodular and diffuse lung disease, this review seeks to outline technologies for improved lung LAC determination.

The required accuracy for lung attenuation estimation will depend upon application. For example, higher accuracy is likely to be needed if quantifying regional uptake in the diseased lung, than for SUV quantification in tumors, assuming that the tumor area has been correctly identified as soft tissue in the MRAC. Where possible, we highlight the implication of LAC variance on SUV variation for either the whole lung, or on a voxel‐by‐voxel basis, using maximum and average values. Quantitative accuracy of the reviewed techniques is predominantly assessed by comparing attenuation estimation with a CT‐based reference standard. The use of non‐FDG radionuclides is also reported where information is available.

## 2 Clinical Motivation

The correct method of attenuation estimation can have notable impact in clinical routine potentially allowing for diminished respiratory artifacts, fewer susceptibility artifacts, improved lesion/disease detectability, and higher accuracy SUV quantification. Inaccurate estimation can complicate the assessment of PET findings and lead to false negative or false positive results. For lung imaging, radiologists often have to diagnose or follow‐up the presence of small pulmonary nodules, for which the correct morphological and metabolic assessment can be overwhelming. Furthermore, new therapies for lung cancer may not change tumor size as a consequence of their action, instead influencing the metabolism of the lesion. Correct metabolic quantification and appropriate attenuation estimation is therefore highly desirable.

**Figure 1:**
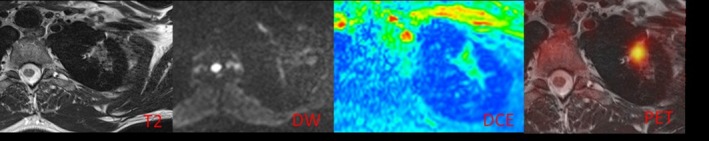
Multiparametric positron emission tomography (PET)/magnetic resonance imaging in a left upper lobe lung mass. T2 weighted and DW (diffusion weighted) images identify the tissue heterogeneity and the increased signal is related to increased tissue cellularity. Neovascularization is seen on the dynamic contrast‐enhanced (DCE) perfusion sequence. Fluorodeoxyglucose PET signal confirms the elevated glucose consumption, a typical pattern of primary lung neoplasms. [Color figure can be viewed at http://wileyonlinelibrary.com]

**Figure 2:**
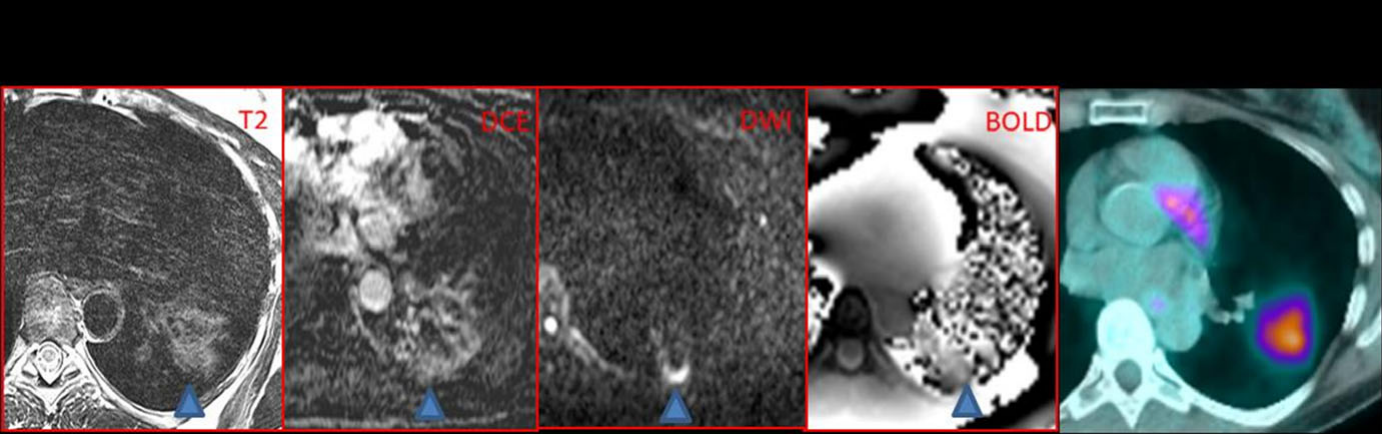
Different sequences provide different information. These parameters can be combined with the results found from positron emission tomography/magnetic resonance imaging (PET/MRI) (right image), or PET/MRI (not shown) to give an “in vivo” biology of tumor heterogeneity. [Color figure can be viewed at http://wileyonlinelibrary.com]

PET/MRI of the lung can be of value for improved patient care compared with PET/CT. Advantages are predominantly the use of an MRI component for dose reduction, tissue characterization, and limited discomfort in patients who require a double exam (PET and MRI). The main application of the MRI component in the lung is the evaluation of soft tissues of the thoracic wall, for example, tumors of the lung apex infiltrating the nervous structures, in the assessment of mediastinal structures, that is, thymoma, thymic carcinoma, or, in all conditions affecting the posterior mediastinum with possible infiltration/assessment of the spine and nervous roots. There are also potential benefits in doing multiparametric PET/MRI, as demonstrated in Figs. 1 and 2.

There are three main domains where accurate AC in a PET/MRI machine is in great demand.

The first area is oncological applications which have primarily been investigated to compare the reproducibility in quantification between PET/CT and PET/MRI. The second aspect is the correct assessment of lung inflammation, both for chronic and acute lung disorders. It is known that PET has demonstrated high standard uptake values (SUVs) of ^18^F‐fluorodeoxyglucose (FDG) in fibrotic lung disease. The assessment of “fibrotic” and “normal lung” becomes essential as it has been shown that regions that appear structurally normal also appear to have higher SUV, suggesting that the disease can already be present, even if not visible, with other modalities (ie CT) and may precede future regions of macroscopic structural change or deterioration of many forms of Interstitial lung disease (ILD).^25^ In this regard, accurate regional quantification appears even more important than in the focal pathology (eg lung nodule, cancer), since the metabolic activity can be used for naïve assessment and longitudinal studies to monitor possible treatments.^26^ In addition, estimation of, and correction for, the regional air fraction, which can be obtained from density maps, is important to be able to characterize different lung diseases.^27^ Finally, the reproducibility of parameters needs to be consistent in the light of further image analysis of radiomic features such as textural information and inaccurate lung attenuation estimation may mislead or influence the values of radiomic features.

It is expected that PET/MRI will improve clinically, in part, because lung MR imaging is advancing to address its main challenges including low signal, image artifacts, and lack of bone‐lung tissue contrast. Lung MRI is likely to be important for both improved LAC estimation and as part of a patient’s general PET/MRI exam. From a clinical perspective, MRI offers promise, particularly given its value in mediastinal mass characterization and cystic fibrosis.^28^, ^29^, ^30^

MRI sequences that improve clinical information and/or aid attenuation correction estimation include spoiled gradient echo (FLASH, SPGR, FFE) sequences, for which two or three dimensional acquisitions can be performed, with or without contrast and/or fat suppression.^31^ They may also include balanced steady state free precession (bSSFP — TrueFISP, FIESTA, BFE) or single‐shot fast spin echo (RARE/HASTE, Turbo FSE) sequences, both of which are routinely used in pulmonary MRI.^31^ Diffusion‐weighted imaging (DWI) may additionally be of value, although its use in lung imaging is less established, for example, as a result of a lack of agreement in the apparent diffusion coefficient (ADC) cut‐off values between benign and malignant masses.^32^ Short tau inversion recovery (STIR) sequences may also be used, such as in imaging lung cancer or mediastinal metastases.^33^ Other advances may arise from multi‐parametric MRI, for example, in the combination of DWI and dynamic contrast imaging (DCE) for lung cancer imaging.^34^ In addition, both motion‐compensated imaging, such as, using self‐navigated sequences or fast Fourier decomposition MRI for noncontrast‐enhanced ventilation and perfusion weighting may lead to higher quality lung imaging.^29^

With the extremely short T2* (of order 0.5–3 ms) relaxation times in the lungs, MRI sequences with near‐zero echo times are very promising.^35^ This gives the potential for detecting lung signal despite the rapid signal decay. Such sequences include UTE, SWIFT, ZTE, WASPI, SPRITE, PETRA, and AWSOS.^35^

In the brain, UTE attenuation estimation has shown success, for example, Jang et al.^36^ established a dual‐echo ramped encoding sequence (acquiring UTE and out‐of‐phase images with the first and second echoes, respectively) to segment fat, water, air, and bone. To transfer such methods to the moving, larger lung FOV, under‐sampling schemes will need to be implemented, and therefore, advanced methods, including parallel imaging, compressed sensing, and machine learning may be necessary.

In the lung, there have been a variety of different UTE/ZTE studies aimed at imaging rather than LAC estimation. As examples, Herrmann et al.^37^ optimized a three‐dimensional (3D) UTE sequence both shortening the repetition time and providing automatic gradient delay compensation. Dournes et al.^38^ produced submillimeter imaging of the lung using pointwise encoding time reduction with radial acquisition (PETRA), and this has also been assessed against a UTE sequence in the lung.^39^ Nazaran et al.^40^ utilized T2* UTE mapping in the lung. Delacoste et al.^41^ performed UTE imaging with advanced reconstruction on cystic fibrosis patients. Feng et al.^42^ undertook four‐dimensional (4D) respiratory motion‐resolved sparse lung MRI. Finally, Jiang et al.^43^ performed 3D image navigation using five‐minute‐free breathing UTE scans to improve resultant image motion robustness.

Such sequences may offer improved lung contrast compared with conventional sequences, potentially allowing for better lung segmentation (and the detection of bone) and improved lung density estimation. However, they suffer long acquisition times and ideally should also be 4D to reduce the effect of MR‐PET spatial mismatch due to breathing.

There are other nonconventional imaging techniques that are being explored. Oxygen‐enhanced MRI can be used to shorten lung tissue, blood, and plasma T1 values, potentially giving ventilation and perfusion MRI weighting,^31^ although lung registration between pre and postinhalation datasets is a challenge.^44^ Signal can be provided from hyperpolarized ^129^Xe or ^3^He gas or inhaled inert fluorinated (^19^F) gas.^44^

## 3 Challenges in the Lung

Performing accurate lung attenuation estimation in PET/MRI is challenging for many reasons.

Firstly, the required LACs are dependent upon tissue electron density but the MRI signal is dependent on tissue proton density and both longitudinal (T1) and transverse (T2 and T2*) magnetization relaxation times.

Secondly, obtaining high‐quality lung MRI can be difficult. Pulmonary MRI inherently suffers from a low signal because the lungs have a low tissue proton density (approximately 10% of other tissues).^31^ In addition, numerous lung‐air interfaces create magnetic susceptibility differences, and this causes the T2* relaxation time to be extremely low, approximately 0.5 ms at 3 T.^31^ Consequently, lung tissue MRI signal decays rapidly following radio‐frequency excitation. Low parenchymal signal can not only make lung density difficult to quantify but may make differentiation of lung and bone (such as within the ribs) unfeasible. This may affect quantification because despite both having low MRI signal for most sequences, air and bone have very different attenuation properties.^2^

**Figure 3:**
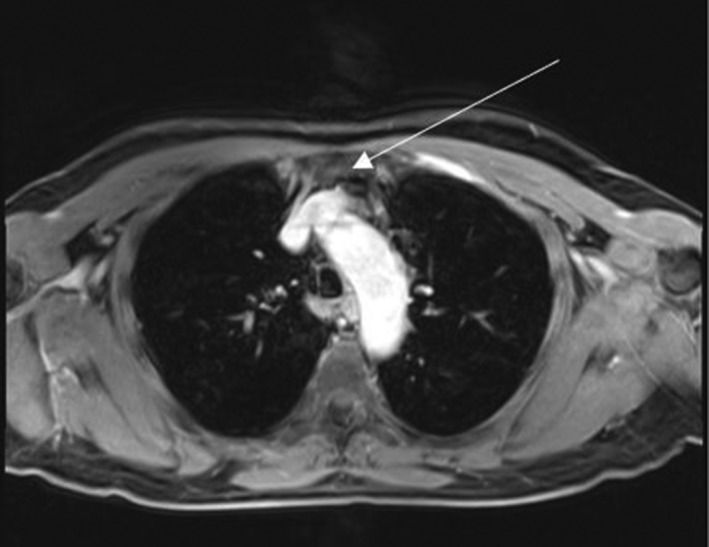
An example of a cardiac ghost artifact propagating into the lung appearing in the anterior portion of the lung (white arrow), with respiratory motion artifacts and pathological signal.

**Figure 4:**
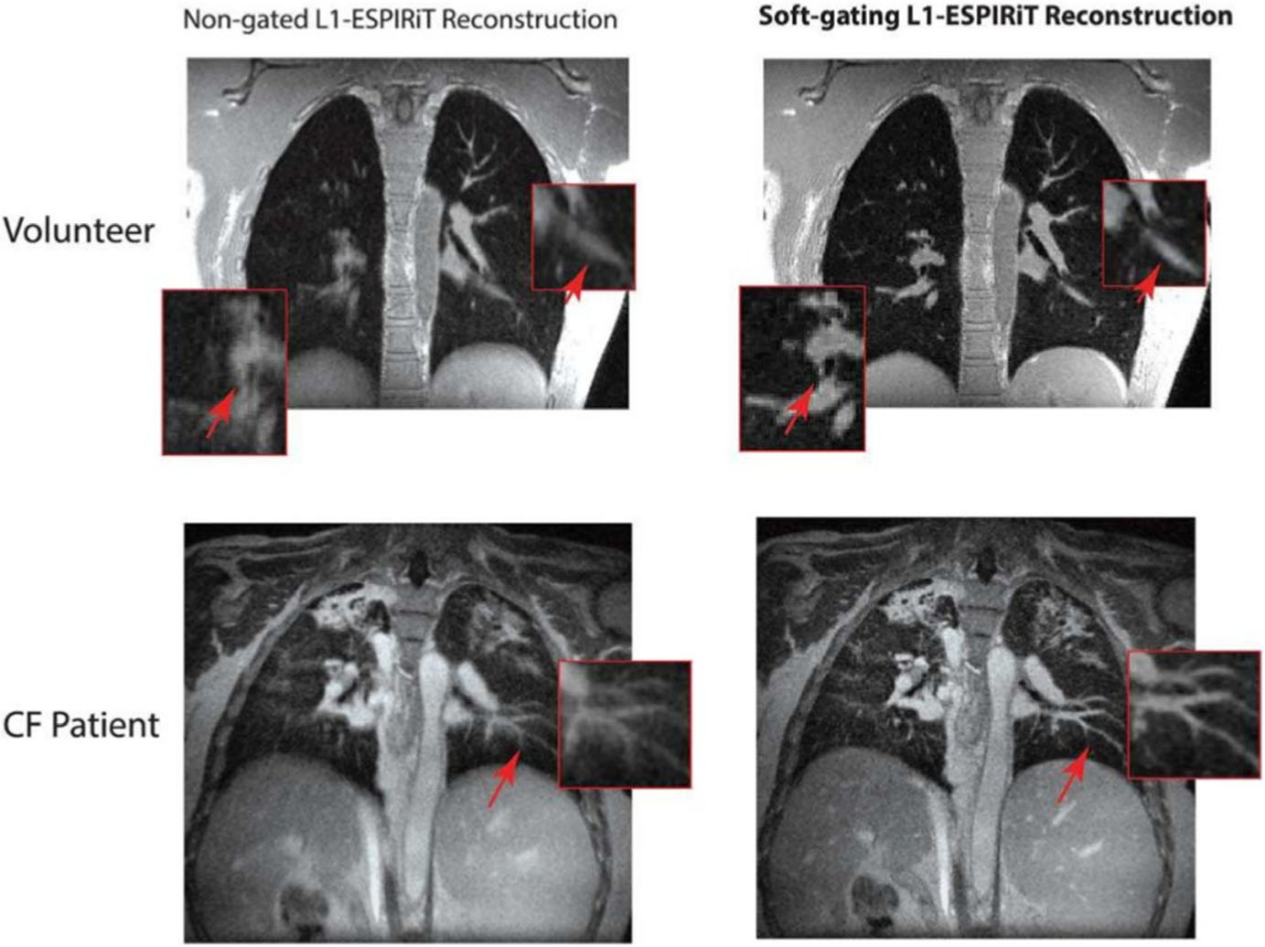
Demonstration of magnetic resonance imaging (MRI) respiratory blurring in both a healthy volunteer (top images) and a patient with cystic fibrosis (bottom images). Left versus right images show the improved performance after considering respiration within reconstruction. The MR images were determined using three‐dimensional ultrashort echo time sequences. Adapted from Jiang et al.^43^ [Color figure can be viewed at http://wileyonlinelibrary.com]

Pulmonary MRI also commonly suffers from image artifacts, including those due to blood flow, a result of the lung's high vascularity, and blurring artifacts from cardiac/respiratory motion.^45^ Example artifacts are presented in Figs. 3 and 4. Inadequate MRI quality can result in segmentation and registration errors that limit the ability to determine continuously valued LACs.

**Figure 5:**
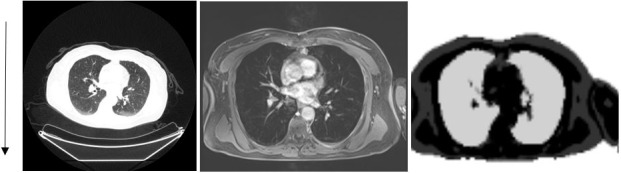
Example computed tomography (CT) [Left], magnetic resonance (MR) [Middle], and MR attenuation correction (MRAC) [Right] images of the lungs. An increase in CT intensity can be seen in the direction of gravity (arrow) but this change in density is not reflected in the MRAC.

Lung LACs are particularly difficult to quantify because they vary, both on a regional basis and from person to person. They show gravitational dependency in the anterior‐posterior direction for a patient lying supine in a scanner, as illustrated in Fig. 5.^21^ Indeed, of the segmented tissue classes used in MRAC, the lungs show the greatest inter‐patient variability, having a reported mean of 0.024 cm^−1^ 46 and standard deviation of 0.004 cm^−1,^ 16, 46, 47, 48 with the lung density (quantified as percentage of pixels in the lung between −750 and −900 HU) varying by up to 30% between patients.^16^, ^49^ As a comparison between tissue types, Bezrukov et al.^50^ determined standard deviations in LACs of 73.47, 26.60, 5.58, and 17.84 cm^−1^ × 10^−4^ for the lungs, fat (hip), muscle, and bone (femur), respectively, in a pediatric set. In an adult set, the authors determined corresponding errors of 46.82, 11.30, 5.21, and 17.03 cm^−1^ × 10^−4^. Intra‐ and inter‐patient variabilities have been suggested to contribute approximately equally to PET lung voxel variation when comparing constant versus continuously valued lung LACs.^51^

A variety of lung density dependencies have been reported in the literature making LAC quantification challenging. Variability in LACs can originate from a variety of different factors including: gender, height, age, pathological conditions, and respiration pattern.^21^, ^49^, ^50^, ^52^, ^53^, ^54^, ^55^, ^56^, ^57^

**Figure 6:**
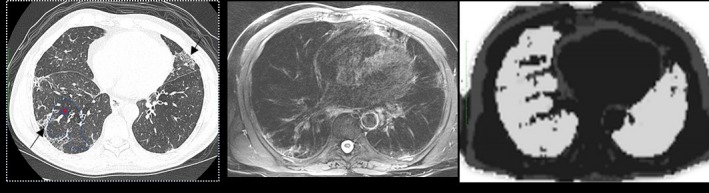
A patient with idiopathic pulmonary fibrosis. High‐resolution computed tomography (CT) [Left] shows increased attenuation areas of reticulation, traction bronchiectasis and ground glass inflammatory changes (black arrows) and low attenuation areas related to obstructive small airways disease (red circle and surroundings). The T2 Blade high‐resolution fat saturation magnetic resonance (MR) image [Middle] acquired at the same level of CT confirms most of the fibrotic changes but is unable to define low attenuation areas due to a lack of signal and diminished spatial resolution (MR imaging — 3 mm, compared with high‐resolution CT — 0.6 mm). The MR attenuation correction map is shown on the right image. [Color figure can be viewed at http://wileyonlinelibrary.com]

Disease can impact lung density,^58^ for example, decreased lung attenuation typically occurs in emphysema or cystic lung diseases, whereas focal increased attenuation occurs in fibrosis.^59^ Often, disease can lead to variable lung attenuation, as shown by example in Fig. 6.

In addition, Karimi et al.^49^ determined that mean lung density is significantly higher in smokers with normal pulmonary function compared to nonsmokers, with females having higher density than males, a variation dependant on height. Bezrukov et al.^50^ also found after comparing lung LACs in pediatric and adult cohorts that lung LAC intra‐patient variation in pediatrics is higher, although adults have a higher mean lung LAC than pediatric patients.

**Figure 7:**
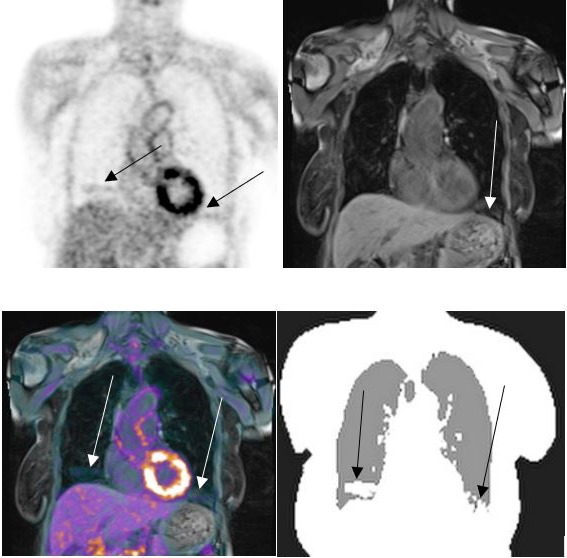
An example of an artifact in the magnetic resonance attenuation correction (MRAC) map. Positron emission tomography (PET) (top left), MR (top right), fused PET/MR (bottom left), and MRAC (bottom right) images are displayed. The inaccuracies appear in both the left and right side of the lung, near the diaphragm and are pinpointed by arrows. [Color figure can be viewed at http://wileyonlinelibrary.com]

Breathing state can also impact lung attenuation. Lung density is lower at end‐inspiration than end‐expiration.^57^, ^60^ Considering MRAC maps, the MRI signal is higher at end‐expiration, as a result of a higher lung proton density coupled with less signal dephasing.^31^ This arises from diminished susceptibility differences across fewer lung‐air interfaces. Furthermore, the difference in duration between the PET and MRI scans can create MRI‐PET tissue mismatches, leading to PET “banana” artifacts near the diaphragm. Figure 7 provides an example of an MRAC artifact in the lower lung. 

A final challenge in lung attenuation is the process of method validation. Comparing different techniques for lung LAC estimation is nontrivial because of multiple other factors. For example, the parameters used in PET reconstruction algorithms, any registration used as part of the attenuation correction calculation, field of view (FOV) truncation compensation, coil attenuation, and nonlung tissue attenuation classes can all influence PET quantification.^19^ Furthermore, given that local PET quantification can be influenced by signal attenuation along the whole line of response^19^ it can be difficult to judge the effectiveness of different methods.

Considering the previously identified challenges, the key requirements of a lung attenuation estimation technique are that it should be robust, patient‐specific, respiratory‐resolved, and clinically feasible. Good practice should be followed in the validation process (as further discussed in Section 5) by considering the effect of other influencing factors, applying well defined metrics (with reference to PET quantification and LAC variation), and comparing to either PET transmission or CT‐based attenuation estimation.

## 4 Past and Present Attenuation Estimation Methods

Attenuation estimation methods for PET/MR can be divided into three categories; segmentation, atlas/mapping, and emission‐based schemes. These are briefly summarized in Table 1, and each type is then subsequently described in more detail in this Section. Techniques that aim to reduce breathing mismatches are also briefly detailed in Section 4.B, which can affect all three categories.

**Table 1:** A brief summary of the three attenuation estimation types.

	Method		
	Segmentation	Atlas/Mapping	Emission
Example advantages	Computationally fast Doesn’t require large cross‐over datasets	Continuous LACs Considers inter‐patient variation	Continuous LACs Computes LACs directly from emission data
Example disadvantages	Mis‐segmentation errors in presence of low signal Single‐valued LACs	Often computationally expensive Requires large database of multi‐modality data	Can suffer from cross‐talk artifacts Tracer dependent
Example citations	Beyer et al.^65^ Arabi et al.^67^ Marshall et al.^68^ Lonn and Wollenweber^66^ Chang et al.^76^ Lois et al.^77^	Arabi and Zaidi^85^ Hofmann et al.^87^ Arabi and Zaidi^73^ Beyer et al.^91^ Marshall et al.^45^	Salomon et al.^100^ Madsen and Lee^105^ Berker et al.^106^ Mehranian and Zaidi^99^ Ahn et al.^110^

### 4.1 Segmentation methods

Segmentation remains the most common form of determining the LACs, and the main vendors use MR images to segment the whole lung and then apply a single‐valued population‐based LAC. The number of tissue classes and choice of LACs depend on the vendor. In the literature, a wide range of lung values have been used (quoted as 0.018–0.027 cm^−1^).^3^ Segmentation techniques benefit from using individual patient data, not requiring large cross‐over datasets (c.f. atlas/learning methods) and being computationally fast. Details of specific vendor MRAC sequences are shown in Table 2. These are all rapid T1‐weighted acquisitions and are not designed to specifically image lung tissue.

**Table 2:** . Details of specific vendor magnetic resonance attenuation correction (MRAC) sequences. Adapted from Beyer et al.^65^ Information relevant to the Siemens sequence is also taken from Paulus et al.^62^

Parameter	Vendor		
	Siemens	Philips	General electric
Version	Biograph mMR (VB20P)	Ingenuity time‐of‐flight (3.2.2)	EMP24.0_EA_1350
Sequence	Dixon VIBE	Multi‐stack spoiled T1w GE	LAVA‐FLEX
Flip angle (°)	10	10	5
TR (ms)	3.6	4.1	4.0
TE (ms)	2.46	2.3	1.7
Matrix	128 × 192	144 × 144	256 × 128
	2.6 × 2.6 mm	4 × 4 mm	4.69 × 4.69 mm
Slice (mm)	2.6	4	2.8
Orientation	Head first supine (coronal)	Head first supine (axial)	Head first supine (axial)
Coils	Surface radiofrequency coil	Built‐in body radio‐frequency coil	Built‐in body radio‐frequency coil
Gated	Yes	No	No
Lung LAC (cm^−1^)	0.0240	0.0219	0.0180
Soft tissue LAC (cm^−1^)	0.1000	0.0950	Variable (0.086–0.100)
Fat LAC (cm^−1^)	0.0854	NA	Variable (0.086–0.100)
Scan time (s) per bed position	19	24	18
Tissue classes	5	3	5

The Siemens mMR method is based on the two‐point 3D volume‐interpolated breath‐hold (VIBE) Dixon sequence for whole‐body segmentation. Here, the sequence exploits the chemical shift difference between fat and water.^61^ Separate “water‐only” and “fat‐only” images allow for a four class (lungs, air, soft tissue, and fat) model. Major bones (including the skull, spine, left/right hip, and left/right upper femur) with continuous LACs are additionally added through a bone segmentation algorithm that uses a set of predefined bone masks and MR images. This is performed through a combination of landmark‐based similarity registration and intensity‐based deformable registration. The method is based on the study by Paulus et al.^62^

The Philips ingenuity time‐of‐flight (ToF) scanner has used a free‐breathing 3D spoiled T1‐weighted gradient echo Cartesian MRI sequence^46^ to segment the lungs. Previously, three tissue classes (air, lungs, soft tissue) have been used for whole‐body PET/MRI. The underlying segmentation method (as described by Schulz et al.^46^) identifies the lungs through a region growing approach together with automatic threshold estimation found from an intensity analysis. To illustrate this segmentation further, in the original study,^46^ each patient firstly had one of their coronal slices automatically chosen based on which had the largest cross section. Using this slice and nearby coronal slices, an intensity threshold was determined using the Laplace histogram, and connected clusters were established by slice restricted region growing. Following this analysis, the authors applied a 3D region growing (intensity threshold based) approach to segment the lungs.

**Figure 8:**
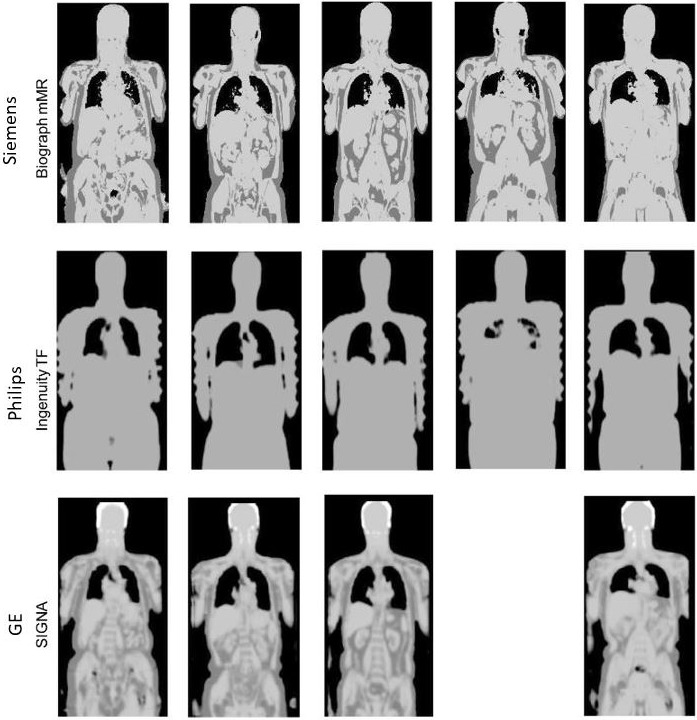
Example magnetic resonance attenuation correction (MRAC) maps from the three major vendors. Adapted from Beyer et al.^65^ Note that the Siemens MRAC images were obtained using the older software version ‘VB18P’, and at this time, bone was not included as a separate tissue class.

The GE scanner has used a 3D dual‐echo RF spoiled gradient echo sequence (denoted LAVA‐FLEX). Four segmentation classes (air, lung, soft‐tissue, and fat) are used (although bone is added in the head^63^). The approach is similar to the Siemens Dixon approach, and the original body segmentation method is described by Wollenweber et al.^64^. Example MRAC maps previously obtained using various vendor software are given in Fig. 8. See Beyer et al.^65^ for further details.

The problem with using single‐valued lung LACs and the variation between different vendor methods has been illustrated by many different studies. Lonn and Wollenweber^66^ measured mean whole‐lung LACs using CT in a patient group, and the range of 0.02 to 0.04 cm^−1^ was shown to substantially change PET activity in reconstruction. Moreover, Beyer et al.^65^ established that mean lung LACs are lower for the SIGNA than the other two PET/MRI scanners. This result may have been influenced by significant intra‐system variation reported in the segmented lung volume of some patients.

Many studies have analyzed lung quantification in and across different whole‐body vendor segmentation methods. While the results do not solely show the effect of using single‐valued lung LACs, the differences reported do strongly suggest the need for improvement.

Arabi et al.^67^ compared three and four class segmentation methods in a 14 oncology patient cohort with cross‐over PET/CT and PET/MRI. Considering six ROIs drawn in the lung, they reported a significant positive mean SUV lung bias of 18.6 ± 15.3% for the three class method and low negative bias of −0.5 ± 13.3% for the four class method, taking PET/CT as the reference standard. Ouyang et al.^51^ also analyzed the potential effect of segmentation using four tissue class segmentation on 23 PET/CT patients. They computed 15.1% root mean square error percentage bias in the lungs.

Other reported results are as follows: Marshall et al.^68^ added bone segmentation to a four tissue class segmentation in a 12 oncology patient cohort, imaged using both PET/CT and Turbo‐FLASH MRI. The atlas used 121 patient CT scans, and the authors found that use of the bone atlas slightly increased mean lung errors from 7.7% to 8% (*P* = 0.002). Furthermore, Bezrukov et al.^50^ compared four class segmentation to an additional bone segmentation (established using both a pediatric and adult atlas). They demonstrated significant variation in the standard deviation of lung SUV (on the order of 15%) for all three methods relative to CT. Wollenweber et al.^64^ also compared four class and three class segmentation in an 18‐person PET/CT and MRI cohort. They reported 14% errors in the lungs. Beyer et al.^65^ showed that total lung integral attenuation values differ by up to 10% across the three systems. While the study was limited in only using four healthy males, the authors note the challenge with multi‐center studies.

**Figure 9:**
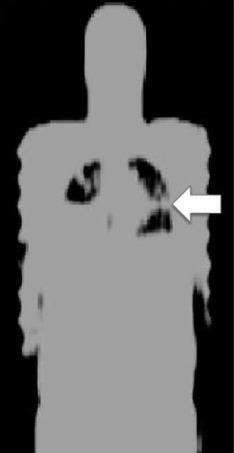
Inaccurate lung segmentation. Adapted from Beyer et al.^65^

In addition to the use of single‐valued LACs, a commonly encountered problem with the above methods are lung segmentation errors. Bone is often not distinguished as a separate class because of its inherently low signal in MRAC sequences.^16^ Moreover, air in the stomach and bowel (along with respiratory and cardiac motion artifacts) can give rise to poor separation of the lungs at the diaphragm.^69^ Mis‐segmentation occurs for all of these reasons, and Fig. 9 shows an example. Keereman et al.^47^ performed XCAT simulations to analyze the effect of segmentation errors. They determined that up to 10% of lung to air misclassification can be tolerated but that the misclassification of lung to soft‐tissue (and indeed lung to bone) leads to significantly greater errors. In a similar study, Akbarzadeh et al.^70^ also undertook non‐ToF XCAT simulations, finding that ignoring bone attenuation increases PET mean relative error by up to 15% for simulated lesions in the body.

**Figure 10:**
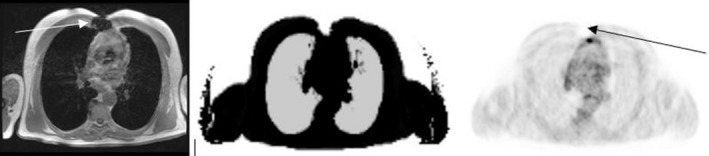
A susceptibility artifact evident in the chest wall on magnetic resonance imaging (Left, white arrow), the attenuation map (Middle), and the positron emission tomography (PET) image (Right). The lung appears continuous with the demarcated air pocket in the reconstructed PET image (black arrow).

**Figure 11:**
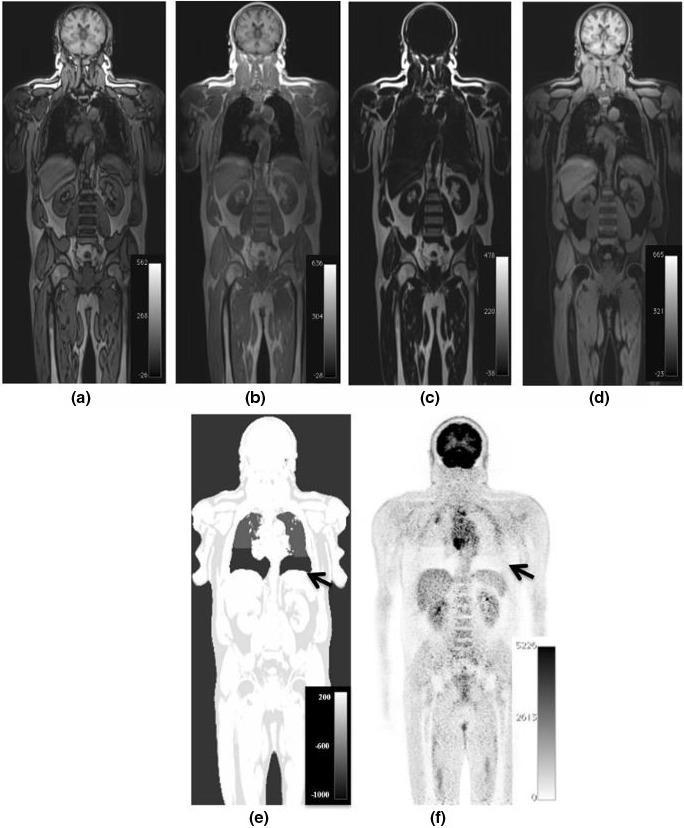
An illustration of tissue misclassification in the lung. The images shown are from a whole‐body [18F]‐FDG positron emission tomography/magnetic resonance imaging (PET/MRI) study of a patient with metastatic lung cancer. Dixon four class segmentation is used with out‐of‐phase (a), in‐phase (b), fat (c), and water (d) MRI given. Figure (e) shows false air tissue assignment to the lower thorax on the MRAC image. Lung attenuation values are assigned correctly to the upper thorax. Figure (f) shows the resultant PET emission image showing an area of severe underestimation of PET activity in the lower thorax, a result of the MR attenuation correction misclassification. Adapted from Keller et al.^135^

Susceptibility artifacts, for example, due to metal, for example, in cardiac stents, can also cause poor segmentation with the lung masks becoming continuous with air outside of the body (Fig. 10). Misclassification artifacts (Fig. 11) also frequently appear, affecting quantification.

**Figure 12:**
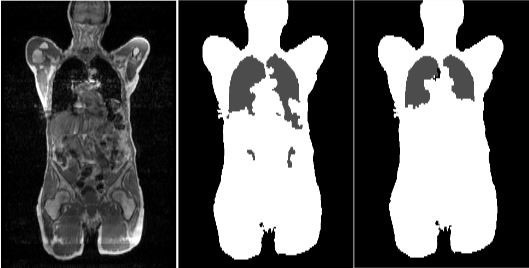
The magnetic resonance image (left), original segmentation (middle), and improved segmentation. Adapted from Hu et al.^69^

A number of studies have aimed to improve the robustness of lung segmentation. Hu et al.^69^, ^71^ used an intensity threshold region growing method with regularization provided by a deformable shape model. Anatomical MR images were established using a T1‐weighted 3D fast gradient‐echo multi‐stack whole‐body sequence. The model consists of a triangle mesh (3000 triangles) established from 20 high‐resolution CT datasets. The model is started by very low threshold intensity‐based lung segmentation. The mesh model is then modified for the patient using an energy minimization scheme. Further postprocessing is then applied to adjust pointed edges, and a region growing technique is applied using both an intensity‐ and distance‐based threshold. Figure 12 shows the improvement gained using a deformable lung model.

Bezrukov et al.^17^ also combined atlas‐based susceptibility artifact correction with standard four class segmentation to reduce the effect of susceptibility artifacts. Furthermore, Shanbhag et al.^72^ used a spatially adaptive phase field segmentation approach and anatomy context‐driven decision making.

**Figure 13:**
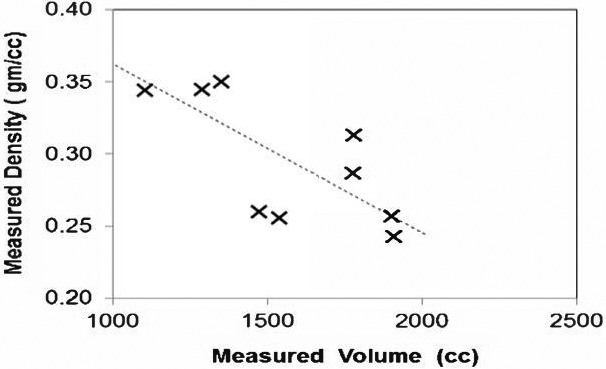
The correlation between mean lung linear attenuation correction and total lung volume. Adapted from Lonn and Wollenweber.^66^

Lonn and Wollenweber^66^ used patient‐specific mean whole‐lung LACs determined after correlating mean LAC to total lung volume (see Fig. 13). Results were compared with those found using generic single‐population LACs, and SUV errors of up to 10% were reported. Reasons for the remaining inaccuracy have been suggested to be a result of neglecting factors including body positioning, pathology, gender, and age.^16^ Mean LAC‐lung volume correlation has also been considered by Arabi and Zaidi^73^ in a multi‐atlas technique, as further discussed in Section 4.C.

In an animal study, Steinberg et al.^74^ segmented the lungs using a T1‐weighted 3D turbo spin echo thrive sequence with fixed and region sized thresholds. Furthermore, Chatterjee et al.^75^ used a multi‐echo ultra‐short echo time (UTE) sequence to generate an MRAC map of a healthy volunteer. Eight echoes were used to segment the lungs, based on a signal model that allowed separation of water, fat and the short T2* lung component. The study was limited by the long‐scan time — ten minutes with respiratory triggering.

**Figure 14:**
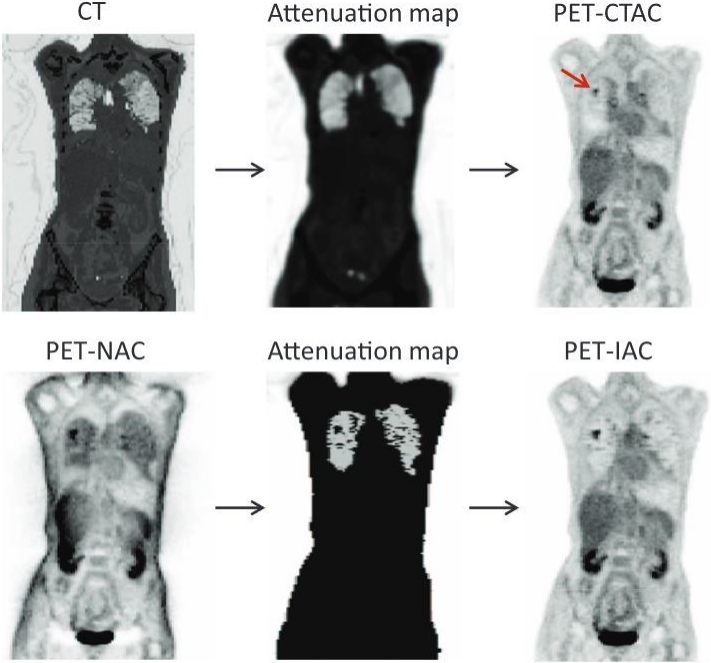
Example computed tomography and nonattenuation‐corrected positron emission tomography segmentation procedures. Adapted from Chang et al.^76^ [Color figure can be viewed at http://wileyonlinelibrary.com]

As an alternative, Chang et al.^76^ performed lung segmentation from non‐attenuation‐corrected PET images in a three stage process. Stage one involved segmenting the whole body in the nonattenuation‐corrected PET image. This allowed for an initial attenuation map to be created that could be used as an input for PET reconstruction. The lungs could then be coarsely segmented in stage two to further update the attenuation map. Stage three further refined the lung segmentation by delineating the heart and liver from the lung contour. Using an iterative process, the method took three times as long as an ordinary PET reconstruction and required some manual interaction. The study also found attenuation correction differences within the lung parenchyma, lung‐liver, and lung‐heart interfaces relative to CT (see Fig. 14).

Finally, the effect of MRI‐contrast agents in LAC estimation still needs to be fully understood in clinical lung MRI, notably considering the influence of contrast on lung segmentation. Martinez‐Möller and Nekolla^4^ state that despite not changing fat/water separation, an increased MRI signal due to the lowering of lung T1 may influence segmentation. Moreover, Lois et al.^77^ reported that ingesting iron oxide‐based oral contrast agent can bias segmented attenuation estimation, for example, resulting in stomach voxels being assigned to the lung, but that the bias is removed with atlas‐based attenuation estimation or use of an alternative contrast agent (pineapple juice). These results were only taken from one patient, so further analysis is required. Other notable nonlung studies are that of Lee et al.^78^ who evaluated the influence of gadolinium MRI‐contrast on PET images for patients with breast cancer, and Borra et al.^79^ who explored the effect of susceptibility effects associated with iron oxide nanoparticles on PET SUV values on the liver, spleen, and pancreas.

### 4.2 Techniques accounting for breathing mismatch

In addition to the previously mentioned techniques, a number of papers have aimed to reduce the effect of breathing‐induced spatial mismatch between MRI‐derived attenuation estimation and PET. These can affect both segmentation methods and the techniques subsequently described in Sections 4.C and 4.D.

Ai and Pan^80^ demonstrated the feasibility of using respiration‐averaged MRI on one patient. Using a two‐dimensional (2D) multi‐slice, multi‐phase spoiled gradient‐recalled echo sequence they acquired 12 consecutive time frames at each slice location to have good coverage over the full respiratory cycle at each slice location.

Kolbitsch et al.^81^ also determined a motion‐corrected attenuation map for use in PET simulations. They acquired MRI data from four volunteers using a 96 s multi‐echo acquisition with a 3D Golden radial phase encoding sampling scheme. Using a self‐gating scheme from 1D foot‐head projections they reconstructed 3D images at different respiratory phases using iterative non‐Cartesian SENSE with a generalized total‐variation constraint in the spatial and temporal directions. They then used motion fields and fat‐water component separation to produce a four‐class motion‐corrected MRAC map. Kolbitsch et al.^82^ have also recently published on MRI‐based attenuation correction for motion‐compensated cardiac PET/MRI.

In contrast, Buerger et al.^83^ computed 4D attenuation maps using two different MRI acquisitions. The first acquisition was a dual‐echo UTE sequence using 0.14 and 4.6 ms echo times and was used to generate a 3D static four class MRAC map. The second acquisition involved generating low resolution (5 × 5 × 1.5 mm) dynamic 3D images with 0.5 s per frame in order to produce 4D AC maps for 4D PET reconstruction. The study was limited to PET simulations and had long MRI scan times (11–16 min).

Finally, Yang et al.^84^ developed a 4D PET respiratory phase‐matched approach with MRI. An undersampled Dixon technique was used to repeatedly acquire 3D data during free‐breathing, and this was reconstructed using compressed sensing. One patient was scanned eight times with ^68^Ga‐DOTA‐TOC PET/MRI and four class MRAC maps were computed using both an external respiratory trace and the self‐gated 4D images. MRI values were converted to continuous CT values using the weighted sum of water/fat values. The authors found that PET tumor uptake values increased in lesions located at the lung‐liver interface for both respiratory correction methods, compared with static MRAC.

### 4.3 Atlas/mapping techniques

Atlas/mapping techniques are an alternative means of estimating LACs in PET/MRI.

**Figure 15:**
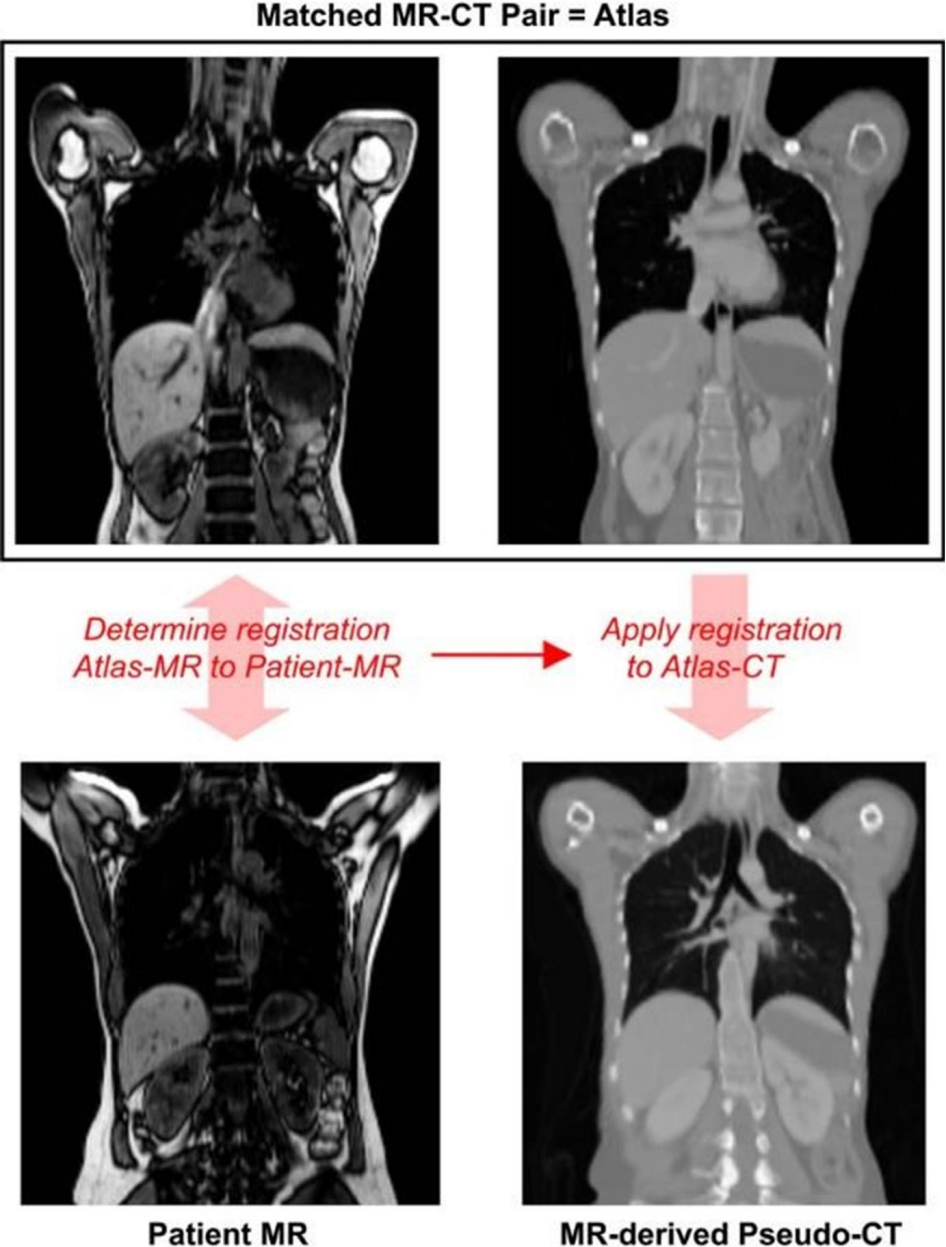
A depiction of atlas attenuation estimation. Adapted from Hofmann et al.^18^ [Color figure can be viewed at http://wileyonlinelibrary.com]

Single or multi‐atlas methods generate LACs in the following way, as depicted in Fig. 15.^18^ An atlas consists of an MR image and either a corresponding PET transmission or spatially aligned CT image. To establish a patient‐specific LAC map, the atlas MR image is first warped to the target MR image, and then the associated deformation (coupled with appropriate LAC scaling) is applied to the transmission/CT atlas in order to generate a patient‐specific LAC map. Multiple atlases can be used to generate the most appropriate patient‐specific attenuation correction image.

Such approaches have the advantage of giving continuously valued lung LACs but due to variability in pathology, the single/multi‐atlas database can potentially fail to yield an accurate patient‐specific LAC map.^16^ Registration suffers from errors due to the nonrigidity of the lung and limitations in the registration method itself, for example, due to local minima in the similarity measure.^51^ Furthermore, multiple registrations can be computationally expensive, affecting the method’s clinical feasibility and can be too dependent on the quality of CT/MRI parameters.^19^

With the long computation times associated with multiple nonrigid registrations, Arabi and Zaidi^85^ aimed to develop a one‐registration approach. Here, precomputed transformations were established between a reference atlas and the remaining atlas images. Registration was then performed between the target image and reference atlas, and using the precomputed transformations, all atlas images were warped to the target image. Twenty‐three patients undertook both PET/MRI and PET/CT, and a leave‐one‐out cross validation approach was employed. Arithmetic averaging and voxel‐wise weighting (VWW) were performed for atlas fusion. Voxel‐wise weighting outperformed arithmetic averaging for both the one‐registration method and direct registration approaches. Both methods reduced lung quantification errors compared with the three‐class segmented MRAC. As an example, VWW following the one‐registration approach, MRAC, and CT exhibited mean lung attenuation values of −794, −770, and −798 HU, respectively. They also determined that the one‐registration technique is approximately a factor of 1/N (N — Number of atlases) faster than the direct registration approach and only suffers from moderately worse errors. Nonetheless, there was still variation in the magnitude of the relative PET error for both the direct registration VWW (6.4 ± 5.4%) and one registration VWW (6.7 ± 6.1%) techniques, relative to PET/CT.

Another variation to increase accuracy and potentially reduce computational cost has been to use sorted atlas datasets. Gender‐dependent, body mass index (BMI)‐dependent, and combined BMI and gender‐independent atlases were previously trialled.^86^ The authors used three BMI categories (Normal, Overweight, and Obese) and separate male/female categories. Due to misregistration in the lung, the authors found PET errors for all methods relative to CT. However, they determined that using categorized atlases gave smaller lung errors than not using categorized atlases, with gender independent atlases giving smaller errors than BMI independent atlases.

Another variation to traditional multi‐atlas techniques has been to additionally incorporate machine learning, so as to map the MRI signal to lung LACs. This has the advantage of potentially increasing robustness to lung anatomical variability and nonrigidity by constraining the registration less.^16^

**Figure 16:**
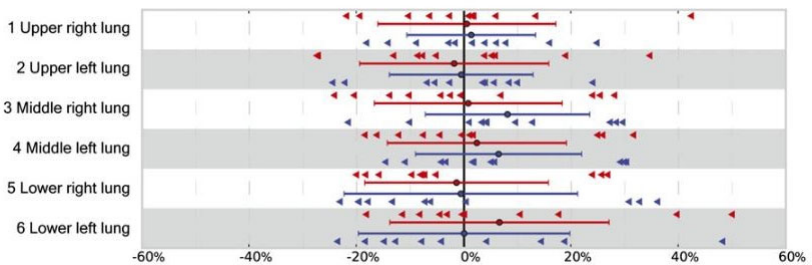
Mean standard uptake value errors in the lung determined by the Hofmann approach (red) and five class segmentation (blue), relative to computed tomography. Adapted from Hofmann et al.^87^ [Color figure can be viewed at http://wileyonlinelibrary.com]

Hofmann et al.^87^ generated continuous lung attenuation maps by adapting their original atlas/machine learning approach.^88^ They used MRI/CT pairs, registering them to the target MR image as in traditional methods. However, they used gaussian process regression to estimate the pseudo‐CT of an unknown voxel considering the local neighborhood. Compared with Hofmann et al.,^88^ their method for the lung used a different registration method, a modified kernel function, and added pre and postprocessing steps. The method was evaluated with a leave‐one‐out cross validation approach. The authors determined mean lung SUV errors (−14.0 ± 11.4%) relative to CT (see Fig. 16). These were slightly worse than a five class segmented CT (13.5 ± 10.7%), potentially due to the small dataset size. The approach has been suggested to suffer from high computation time.^16^

**Figure 17:**
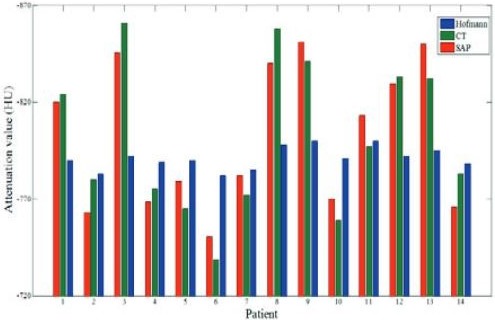
Mean lung linear attenuation corrections on an individual patient basis determined from computed tomography (green), the Hofmann approach (blue), and the Arabi approach. Adapted from Arabi and Zaidi.^73^ [Color figure can be viewed at http://wileyonlinelibrary.com]

An updated method has been given by Arabi and Zaidi.^73^ For nonlung, these authors sort atlas images based on voxel‐wise local normalized cross correlation. This is to find the best match to the target image, as was originally suggested for the brain by Burgos et al.^89^ More importantly for lung, Arabi and Zaidi^73^ used a 50 patient CT dataset to incorporate a mean lung LAC‐total lung volume correlation into the Gaussian process regression kernel, possibly based on the observation of Lonn and Wollenweber^66^ (see Section 4.A). Arabi and Zaidi^73^ determined that with 14 patients, whole‐lung mean SUV bias fell from 8.9% following Hofmann et al.^87^ to 4.1%, relative to PET/CT. These results are shown in Fig. 17. The study found that both atlas/machine learning methods had reduced SUV mean error in the lungs, compared with three class MRAC. However, computation time was long per target image (1100 min) owing to the multiple registrations and Gaussian process regression training.^90^

As an alternative, several studies have attempted to perform MRI‐CT lung mapping, from which LACs can be generated. Beyer et al.^91^ employed histogram matching. Ten oncology patients had PET/CT and contrast‐enhanced transverse T1‐weighted VIBE MRI torso scans. The MRI and CT pairs were co‐registered using both mutual information and elastic regularization. Histogram matching involved (a) using the individual pixel intensities in the MR and CT 2D images to form intensity histograms, (b) summing the 2D image histograms to form a 3D cumulative histogram, (c) finding matching pairs of CT and MRI intensities for which the cumulative intensities are equal, and (d) generating a MRI‐CT mapping table from the matched pairs. The study found PET errors determined in the lungs were likely due to the lack of anatomical information used in the mapping coupled with poor lung MRI contrast and misregistration.

**Figure 18:**
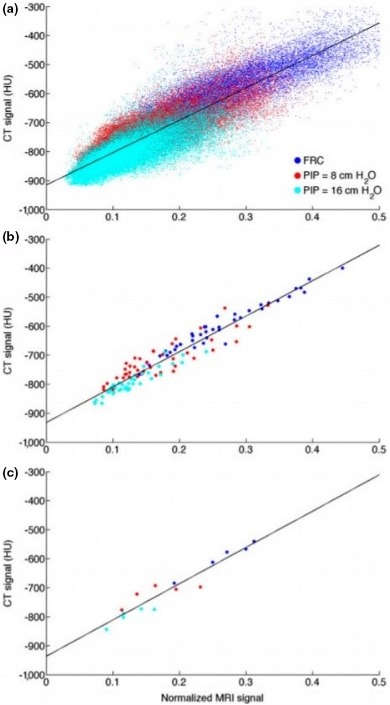
Computed tomography lung signal as a function of magnetic resonance imaging, derived using individual voxels (a), mean signal intensities of coronal slices (b), and whole‐lung mean signal intensities (c). The three colors represent different breathing states. Adapted from Marshall et al.^45^ [Color figure can be viewed at http://wileyonlinelibrary.com]

Alternatively, Marshall et al.^45^ performed an MRI‐CT mapping animal study, with cross‐over PET/CT and two‐dimensional turbo fast low‐angle shot (Turbo‐FLASH) MRI. A three‐class tissue model was used where the lungs were segmented using voxel seeding based on an intensity threshold. The lung pair images were co‐registered, and the intensity values were then correlated using (a) individual voxels, (b) mean signal intensities of coronal slices (to maintain dorsal to ventral density gradients), and (c) whole‐lung mean signal intensities. PET reconstructions were then performed using the derived mappings on the MR images and compared with reconstructions that assumed constant lung CT attenuation. Three respiratory states were considered, and Fig. 18 shows the established correlations.

Marshall et al.^45^ determined that while all three mappings led to improved lung PET quantification compared with using constant lung LACs, the individual voxel mapping gave highest accuracy. Nonetheless, the lungs were consistently mis‐segmented by approximately 15% in all respiratory states, and despite segmentation being independent of the mapping procedure this would have affected the mapping results. This study was also limited by being on animals who underwent external ventilation. Furthermore, such a mapping generally requires a standardized protocol and is influenced by respiratory motion, diamagnetic susceptibility, and flow‐based artifacts.^16^

### 4.4 Emission‐based schemes

Emission‐based schemes estimate the attenuation image directly from the emission data and are particularly promising for overcoming the quantification errors induced by conventional segmentation‐based attenuation estimation methods.

Such schemes can be broadly divided into two categories: analytic and iterative reconstruction types. Analytic methods estimate the attenuation distribution without having to reconstruct an activity map and rely on the consistency conditions of the attenuated Radon transform.^92^, ^93^ Iterative methods aim to iteratively reconstruct the attenuation together with the activity distribution of the object, most commonly with simultaneous maximum likelihood reconstruction of activity and attenuation (MLAA), possibly combining *a priori* knowledge about absolute attenuation values.^94^ Iterative methods potentially benefit from modeling the underlying physics of the process and take into account the Poisson nature of the data. However, the improvement on analytic approaches comes at the cost of greater computational demand.

The MLAA method consists of an alternating optimization approach in which the activity distribution and attenuation distribution are updated repeatedly. At each step, the problem can be traced back to either emission^95^ or transmission tomography^96^ reconstruction. However, the joint estimate of activity and attenuation has been found to be highly ill‐posed. In fact, it suffers from the so called cross‐talk artifact, where errors in the activity estimate are compensated by errors in the attenuation map. The availability of time‐of‐flight (ToF) PET allowed this limitation to be reduced, leading to a more stable solution of the joint‐problem.^97^ Nevertheless, the attenuation image can only be determined up to an unknown offset, resulting in an unknown scaling factor in the reconstructed activity image.^98^ Overall, the performance of ToF‐MLAA algorithms strongly depends on ToF timing resolution.^99^ With the advent of ToF‐PET/MRI scanners, Salomon et al.^100^ incorporated segmented MRI information into the ToF‐MLAA estimation of attenuation coefficients. The segmentation of MRI data is used to distinguish different regions for the subsequent update of the attenuation values. The attenuation coefficient of each region was initially prescribed the value of water at 511 keV. Subsequently, ToF‐MLEM (Maximum Likelihood Expectation Maximization) and a gradient‐ascent‐based algorithm were used to jointly update the activity and attenuation values for each region. The results showed a considerable reduction in noise and cross‐talk artifacts. To reduce the inherent cross‐talk of MLAA, Atibi and Rezaei^101^ recently proposed to incorporate a tissue prior atlas (TPA) and a Gibbs prior, representing the attenuation coefficient estimates as a mixture of pseudo‐Gaussian distributions. The algorithm outperformed the MR‐MLAA algorithm proposed by Heusser et al.^102^ In addition, Rezaei et al.^103^ evaluated MLAA in ToF PET using a whole‐patient dataset, where the scaling problem in MLAA was resolved for the evaluation by imposing the total activity of the MLEM reconstruction. The authors reported a difference of −7.5% ± 4.6% between MLAA and MLEM after averaging on several regions of interest, including within the heart, bladder, liver, and a lumbar vertebra. The same group has recently evaluated MLAA in ToF PET for brain applications.^104^ A uni‐modal soft‐tissue intensity prior was used in this work to constrain the reconstructed attenuation. Results showed that the activity image reconstructed with MLAA is comparable to an emission reconstruction with a ZTE‐based AC, as well as CT‐based AC.

A limited number of studies focus on the derivation of patient‐specific lung attenuation coefficients in emission‐based attenuation estimation methods.

Madsen and Lee^105^ proposed to use the consistency conditions to refine the lung boundary estimation, previously obtained from a lung atlas. This is necessary for accurate lung‐liver delineation. The (non‐TOF) MLAA framework was then used to estimate lung (and other whole body) attenuation coefficients. This method was only applied to studies where the arms were not in the field of view (FOV). As stated, arms in the field of view might increase the difficulty for determining body contour boundaries.

Berker et al.^106^ explored a constrained ToF‐MLAA algorithm for estimating mean lung LACs using a five‐class whole‐body MRAC map as input. The mean lung LAC value was initially assigned a homogeneous value of either 0 or 0.05 cm^−1^, and the value was subsequently updated while keeping the remaining tissue LAC values constant. The results obtained with Monte Carlo simulations showed high PET quantification bias, possibly due to out‐of‐FOV coincidences, and the unsolved scaling problem in the MLAA algorithm.

More recently, Mehranian and Zaidi^99^ proposed to derive continuous and patient‐specific lung LACs from ToF‐PET emission data using the MLAA algorithm. The approach consists of a voxel‐wise estimate of the lung LACs, with a consequent improvement over previous methods that only estimated mean lung attenuation values. In this work, only the lung values are updated, while the attenuation coefficients of the other class MRAC maps are assumed to be known. The objective function used in reconstruction consists of the Poisson log‐likelihood of the data, a Markov random field smoothing prior, and a Gaussian prior. For the lung, the Gaussian function was centered at the attenuation coefficient expectation value of a patient population. The results showed that the proposed ToF‐emission‐based algorithm can recover lung density values, compensate for respiratory‐phase mismatch between PET and CT, and reduce average lung relative errors, compared with the MRAC method. The approach assumes that MRI information is sufficiently reliable in other nonlung regions. However, this is not necessarily the case, since potential segmentation and classification errors in addition to uncertainties in tissue attenuation values remain to be fully addressed.^107^

It has also been shown that TOF PET reconstruction is less sensitive than non‐TOF to mismatches in the PET and CT acquisitions.^108^ Similarly to density changes in PET‐CT, the same consideration would apply for wrong attenuation assignment by segmentation methods in PET‐MRI. In this regard, Mehranian and Zaidi^109^demonstrated that TOF‐PET can significantly reduce quantification error in the lung and bone tissue, due to errors in the MRAC. In the study, the non‐TOF MRAC method achieved an error of −3.4% ± 11.5% in the lungs and −21.8% ± 2.9% in bones, whereas TOF MRAC reduced the error to −2.9% ± 7.1% and −15.3% ± 2.3%, respectively.

Both non‐FDG and FDG‐based methods involve the detection of 511‐keV annihilation photons, and therefore, attenuation is not specifically affected by the choice of radionuclide. However, the distribution in both the activity and target to background does vary between different radiopharmaceuticals/tracers. This will affect the performance of any emission‐based method, including MLAA. For instance, for 2D TOF PET Defrise et al.^97^ have shown uniqueness (up to a global scale factor) of the projections of the attenuation map for all lines of response with nonzero activity only. This can lead to problems with the attenuation map estimation when the activity distribution does not match the spatial extent of the mu‐map. The use and comparison of non‐FDG radionuclides in emission‐based studies has been extremely limited. One study by Ahn et al.^110^ evaluated a joint estimation of activity and attenuation algorithm using an MR‐based prior both with clinical whole‐body non‐FDG (^68^Ga‐DOTATOC and ^18^F‐Fluoride) and FDG PET/MRI data. A comparison between FDG and non‐FDG performance was not possible given that no patient received more than one tracer type. However, the authors did report better lung delineation using both FDG and the specific and low background uptake non‐FDG tracers, relative to segmented MRAC.

To finish this Section, recent work has indicated that MLAA can be sensitive to errors in the system modeling^111^ including timing calibration. Methods to compensate for this are still under development.^112^

## 5 Potential Future Strategies

Space and complexity constraints make it seem unlikely that CT or transmission sources will be integrated within clinical PET/MRI scanners for use in routine imaging sessions. In the absence of this direct measurement, the attenuation information has to come from either the available PET signals, MRI signals, prior knowledge, or a combination of techniques. Based on our experience, this section outlines seven possible strategies for lung attenuation estimation and validation.

### 5.1 Dynamic PET data

PET/MRI offers the potential advantage of simultaneous dynamic PET and MRI data acquisition. The information from multiple dynamic PET time frames could then be used for improved determination of the LAC estimate. Previously, Rashidnasab et al.^113^ have extended the MLAA algorithm to dynamic MLAA (dMLAA), to jointly reconstruct the activity distribution and a single attenuation map from dynamic PET data. The proposed dMLAA alternately estimates the activity distribution for each time frame, holding the attenuation map at its current estimate, and then updates the attenuation map using a maximum likelihood for transmission tomography (MLTR) approach. The method has been evaluated for dynamic brain PET data and results show that dMLAA improves the reconstructed attenuation map compared to using a single ToF emission frame. Currently, despite the additional dynamic information, non‐ToF reconstruction still suffers from cross‐talk but in the future the concept may be applied to improve LAC estimates.

### 5.2 Scattered PET data

Motivated by the fact that attenuation in PET is mainly due to Compton scattering, and that scatter events represent up to 40% of the total recorded coincidences, several groups have considered the possibility of using scattered emission data as an additional source of information^114^, ^115^ to overcome reconstruction ambiguities in MLAA, especially without ToF data. These attempts share similarities with SPECT studies,^116^, ^117^, ^118^ and extend the earlier suggestion of using individual photon energy information.^119^ The idea of deriving quantitative information from scatter essentially relies on the possibility of estimating scattering angles through photon energy measurements, although this is limited by the energy resolution of the PET detectors.

Previously, Berker and Schulz^120^ developed the so‐called “scatter‐to‐attenuation reconstruction algorithm” (S2A). Their study showed that adding scatter information improves the quality of the reconstructed activity distribution. This work relies on the idea that in PET, given detector locations and the corresponding measured energies for a coincidence, the set of possible scattering locations can be described geometrically. However, this is only straightforward if energy measurements of the scattered photons are perfect, as assumed in this paper. The method relies on a “scatter‐to‐attenuation” (S2A) back‐projection approach. Corrections for effects such as detector geometry and scatter probabilities are applied as a multiplicative constant. The work provides a proof‐of‐concept investigation into the reconstruction of the attenuation image from only the scattered data (assuming a known emission image).

In subsequent years, the group moved from a heuristic back‐projection method to a gradient‐ascent optimization algorithm where the Poisson log‐likelihood for data in multiple energy windows is maximized for the attenuation map with a known emission image.^121^ Results showed that this method outperforms S2A, although further development is necessary to model scatter beyond two dimensions and consider the dependency of attenuation on energy.

The energy resolution of current PET/MRI detector systems is only around 15%–16% and when dealing with scattered data, there is a need for more accurate energy resolution modeling. Simulation work by Brusaferri et al.^122^ indicates that it is possible to obtain an attenuation map from scattered PET data only (using an energy resolution equal to 16%) even without ToF, albeit at low spatial resolution and assuming that the emission image is known. Their energy‐based attenuation reconstruction algorithm extends current methods by incorporating multiple energy window measurements, accurate energy resolution modeling, as well as the energy dependency of attenuation. A future extension might be the incorporation of MR information to improve the reconstructed attenuation map.

### 5.3 Using single event data to determine the attenuation sinogram unknown constant

In addition to the potential use of energy information, it seems logical to also try to use data from singles events. These could help break the symmetry between activity and attenuation factors. An iterative algorithm has been developed^123^ to reconstruct the activity and attenuation distribution utilizing ToF data and single events. The algorithm consists of updating the activity distribution using ToF‐MLEM and updating the attenuation map using MLTR with non‐ToF data. Subsequently, constant coefficients in both activity and attenuation distribution were corrected with single event data information. There is potential for future work to take forward these 2D simulations that used noise‐free data where both scatter and “randoms” information were assumed to be known.

### 5.4 MLAA MRI priors

PET/MRI can provide MR data that is spatially and temporally aligned with the PET acquisition and thus might be useful as subject‐specific priors for the PET reconstruction.

Previously, Mehranian et al.^124^ proposed the incorporation of a multi‐parametric anatomical‐functional (MR‐PET) prior. The regularization term consists of a local joint‐entropy penalty function that relies on both PET and MRI information. As the authors stated, the work suggests that the scaling problem of the MLAA algorithm can be addressed by imposing MRI spatial constraints on the attenuation estimate. The approach has been further assessed in a MRI‐guided MLAA.^125^ Results show that PET images are simultaneously corrected for both attenuation and the partial volume effect (PVE). The algorithm outperforms MLAA, enabling noise suppression, and enhancing boundaries. Nonetheless, these were brain studies, and further investigation is needed for the lungs. Furthermore, both approaches were limited to ToF PET. Another relevant study is that of Mehranian and Zaidi^126^ who proposed a MRI‐driven MLAA algorithm in whole‐body PET/MR imaging. MR spatial and CT statistical constraints were imposed in the form of a constrained Gaussian mixture model and a Markov random field smoothness prior. When compared with other MLAA algorithms, cross‐talk and scaling problems of activity and attenuation were reduced.

### 5.5 MR‐CT learning

Machine learning is making rapid advances in many areas. Viewed as a technique for learning a function from training data, these methods offer the possibility of learning the relation between the MR image and the CT and hence the LAC from a bi‐linear scaling of the predicted CT. It has been shown by Marshall et al.^45^ that the MR signal does exhibit some correlation with CT signal. However, there is no clear direct mapping from MRI to CT and this is where machine/deep learning may be able to learn additional information and advance the field.

Learning approaches have shown success in nonlung attenuation correction, and therefore, they may be of value in the lung. For example, Nie et al.^127^ developed a three dimensional fully convolutional neural network approach to generate pseudo‐CT images from MRI data in a patch‐based manner. The authors tested their network on a pelvic dataset and, their method outperformed atlas, structured random forest, and auto‐context techniques. Deep learning has also been applied to lung pattern classification for interstitial lung diseases (ILDs). Using 120 CT scans, Anthimopoulos et al.^128^ followed a convolutional neural network approach to categorize ILD patterns into seven types, determining an 85.5% classification success rate. Given that many more studies that have been published for nonlung attenuation correction, it is recommended to analyze the previously developed methods in order to address their suitably for lung MRAC application.

In addition, machine/deep learning may be used in combination to improve either segmentation or emission‐based attenuation estimation techniques. As an example of this, albeit not specifically targeted to attenuation estimation, Gong et al.^129^ trained a deep residual convolutional neural network using interpatient information to improve upon a PET iterative reconstruction framework. They solved a constrained optimization problem using an alternating direction method of multipliers algorithm using six patient PET datasets. The proposed method outperformed neural network denoising and conventional penalized maximum likelihood methods in the lung, demonstrating higher lesion contrast to noise ratios. While the approach was solely targeted to PET images, the authors do state that the model can be applied in either a CT or MRI framework.

In the lung, the accuracy of attenuation correction learning will depend on the quality of the underlying image dataset. When paired MR‐CT images are used, learning methods have been dependent on the registration quality of the dataset images. Nonetheless, the use of unpaired data may be feasible, as recently demonstrated by Wolterink et al.^130^ The authors developed a generative adversarial network approach, generating pseudo CT images from two dimensional MRI brain data with a 24 patient cohort.

Challenges may also arise where MRI lung signal is poor, resulting in the network having to focus on low level features, for example, points between images or edges. Here, transfer learning may be beneficial because using an already trained model may operate as a good starting framework for subsequent lung MRI‐CT training. This may also be advantageous where small datasets exist. In any approach, the performance of different MRI sequences (T1/T2/PD/UTE/ZTE, etc) and the various scan parameters (resolution, acquisition time, signal to noise ratio, etc) should be assessed. The size of the required dataset also needs to be better understood. Nevertheless, the rapid advances in the field of deep learning might make it possible to incorporate prior knowledge from previous data and physics understanding of scattering and singles to create subject‐specific LAC estimates. A notable study is that of Hwang et al.^131^ who assessed the ability of deep convolutional neural networks within MLAA to overcome limitations of cross‐talk, slow convergence speed, and noisy attenuation maps. The authors applied the method to a clinical brain dataset and reported decreased noise and increased uniformity within attenuation maps and also diminished cross‐talk problems for the combined deep learning method compared with standard MLAA”. Similar results might be expected within the lung, and moreover, the use of deep learning to aid reconstruction might also be expected to contribute to lung reconstruction in the future.

### 5.6 Registration and deformation of an existing attenuation map

If an attenuation map is available from a previous CT study, a possible strategy is to register this to the PET and/or MR data. In contrast to brain PET/MR where rigid registration can be used, this needs deformable registration for the thorax. Nonattenuation‐corrected PET images might not contain sufficient information for successful registration in the thorax, therefore MR images can be used. However, this can lead to problems with mismatched breathing states between CT, MR and PET. Recent methods, including maximum‐likelihood activity reconstruction and attenuation registration (MLRR), attempt to overcome this problem by estimating the deformation field based on the PET data.^103^, ^132^ However, these approaches currently do not take density changes during respiration into account. Moreover, it is difficult to see these methods becoming widespread in clinical practice due to the need for a previous scan and the associated data management issues even if it exists.

### 5.7 Method validation

A remaining challenge lies in how best to assess potential lung attenuation estimation methods, both for the inherent accuracy of the LAC estimate, and the impact upon diagnosis or treatment monitoring. The lack of available realistic phantoms for PET, MRI, and CT limit their use in attenuation estimation, although for example, PET‐CT‐MRI phantoms have been proposed containing four representative tissue types, with the lung section being based on a pig lung lobe.^133^ Patient cross‐over studies where both PET/CT and PET/MRI data are available from the same patient are informative, though have limitations in terms of accuracy of image registration and lung density changes due to differences in breathing and patient repositioning. Furthermore, the literature has mixed opinion on whether PET transmission or CT‐based attenuation estimation should be considered as the reference standard. Inaccuracies in the LAC estimate will affect the PET quantification but the impact of this is application specific. There may be higher LAC accuracy requirements for applications requiring an absolute SUV value, for example, the monitoring of a treatment response, or decisions based upon a threshold SUV, compared to applications where a relative change in SUV is adequate.

Additional variability can arise from different PET reconstruction parameters, registration algorithms, FOV truncation compensation,^134^ MR‐coils, SUV error types (mean vs maximum or whole‐lung vs a voxel basis), and patient diseases. A better consensus and more lung‐specifically targeted studies would be of great benefit for future applications of lung PET/MRI.

## 6 Conclusions

Despite the potential of PET/MRI for whole‐body imaging and pulmonary studies, lung attenuation estimation has been an under‐appreciated problem. This review has identified the key challenges associated with determining lung attenuation maps. It has highlighted some of the issues by examining past and present methods for estimating lung linear attenuation coefficients (LACs). Current commercial practice is to employ single‐valued population‐based LACs following segmentation of MR images. The use of single‐values impacts PET quantification because underlying lung LACs vary both on an individual basis and from person to person. Commercial roadmaps are not usually made public, but it is hoped that vendors will be able to implement techniques that lead to improved lung quantification. With active developments taking place, it is difficult to predict the techniques that will be adopted in the future, though a combination of improved MRI acquisitions and machine learning or MLAA approaches are currently looking promising. Hopefully advances will lead to clinically available systems that can offer information with high diagnostic or prognostic value for patients.

## Conflict of Interest

The authors received grant funding from Siemens Healthineers for a lung attenuation correction project. They also receive GE Healthcare and GlaxoSmithKline funding for unrelated lung quantification research.
